# Disseminated herpes simplex virus type 2 infection manifesting as cutaneous vasculitis: A case report

**DOI:** 10.1016/j.jdcr.2025.09.004

**Published:** 2025-09-19

**Authors:** Yen-Yi Sung, Jau-Yu Liau, Chia-Yu Chu

**Affiliations:** a Department of Dermatology, National Taiwan University Hospital and National Taiwan University College of Medicine, Taipei, Taiwan; b Department of Pathology, National Taiwan University Hospital, Taipei, Taiwan

**Keywords:** cutaneous vasculitis, disseminated herpes simplex virus infection, herpes simplex virus type 2

## Introduction

Herpes simplex virus (HSV) type 2 infection is frequently seen in daily practice and typically shows grouped vesicles or erosions involving the genital and anal area. However, the clinical presentation of disseminated HSV infection varies, and clinicians should be especially aware of its manifestation in immunocompromised patients.^1^ In this report, we describe a case with cutaneous HSV-2 infection presenting as generalized vasculitis.

## Case report

**Fig 1:**
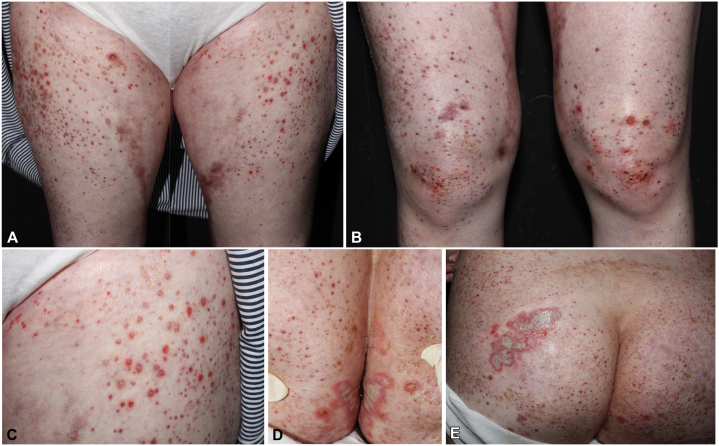
**A-C,** Erythematous, purpuric papules and plaques on the lower extremities with scattered punched-out ulcers. **D** and **E,** Grouped ulcers on the intergluteal folds and left buttock.

**Fig 2:**
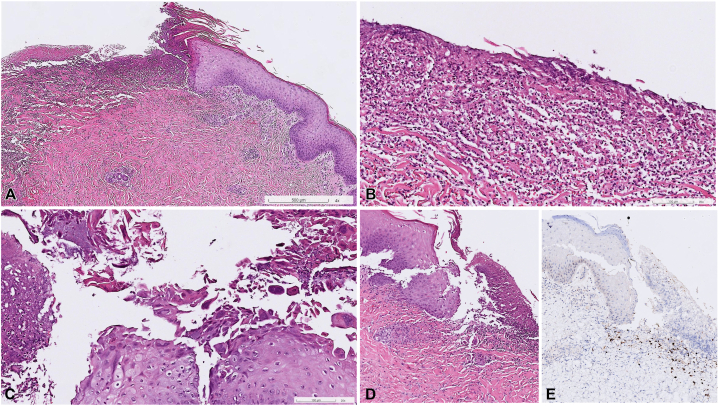
Histopathologic features of the skin biopsy from the right knee. **A** and **B,** Epidermal necrosis with neutrophil infiltration. **C,** Viral cytopathic effects with multinucleation, chromatin molding, and margination. **D,** Leukocytoclastic vasculitis in the dermis with vessel wall disruption, erythrocyte extravasation, and fibrinoid necrosis. **E,** Herpes virus infection confirmed by positive herpes simplex virus immunohistochemical stain (**A-E,** hematoxylin-eosin stain; original magnification: **A,** ×40; **B** and **C,** ×200; **D** and **E,** ×100).

A 43-year-old female patient presented to our dermatology clinic with a 2-week history of a sudden outbreak of a generalized painful rash. She had been otherwise healthy until being diagnosed with idiopathic urticarial vasculitis at another hospital 3 months ago and was under treatment with prednisolone, colchicine, and azathioprine. On physical examination, numerous erythematous, purpuric papules and plaques were observed on her arms, trunk, and lower extremities. In addition, there were grouped erosions and ulcers on erythematous bases with pus discharge on the intergluteal folds and left buttock (Fig 1). A skin biopsy obtained from the right knee disclosed epidermal necrosis, with neutrophil infiltration (Fig 2, *A, B*), viral cytopathic changes with multinucleation, chromatin molding, and margination (Fig 2, *C*), suggesting herpes infection. Leukocytoclastic vasculitis in the dermis, with disruption of the vessel wall, erythrocyte extravasation, and fibrinoid necrosis, was present (Fig 2, *D*). Immunohistochemical staining highlighted the presence of HSV (Fig 2, *E*), whereas cytomegalovirus and periodic acid–Schiff staining showed negative results. Polymerase chain reaction analysis of the buttock erosions also confirmed HSV infection. HSV-2 was isolated from the buttock ulcers. Serum levels of antinuclear antibody, anti-double-stranded DNA antibody, anti-phospholipid antibody, anti-cardiolipin antibody, p-anti-neutrophil cytoplasmic antibody, c-anti-neutrophil cytoplasmic antibody, anti-extractable nuclear antigen, lupus anticoagulant test, and complements 3 and 4 were all within normal limits. Serum protein electrophoresis showed a nonspecific pattern. Her data from the previous hospital showed unremarkable results for the antistreptolysin O test, hepatitis panel, rheumatoid factor, and cryoglobulin. The patient also denied recent infection episodes. HSV-induced vasculitis was indicated.

**Fig 3:**
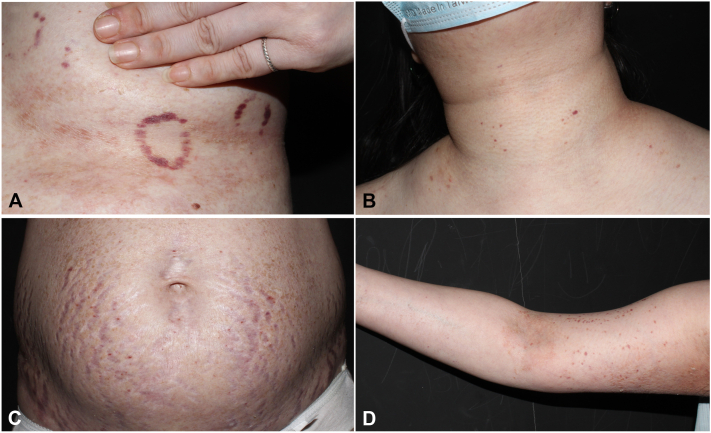
Palpable purpuric papules and plaques on **(A)** left submammary area; **(B)** neck and upper portion of the chest; **(C)** abdomen; and **(D)** upper portion of the left arm.

**Fig 4:**
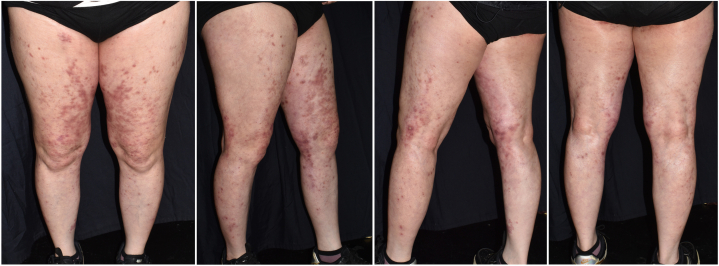
Vasculitis lesions resolved with prominent postinflammatory hyperpigmentation on the lower extremities.

Oral valacyclovir 500 mg twice daily was prescribed for 10 days, but new vasculitis lesions emerged after discontinuation (Fig 3). Meanwhile, we discontinued the patient’s immunosuppressants and gradually tapered prednisolone to 5 mg/d in the following 10 days. Oral valacyclovir dosage was escalated to 1000 mg 3 times a day for another 3 days because of the inadequate response of the immunocompromised patient. The patient was discharged from the hospital on day 17 with significant resolution of the rash and healing of the ulcers. All the skin lesions were resolved completely within 7 months with continuous use of valacyclovir 500 mg daily without immunosuppressant treatment (Fig 4). Antiviral dosing was adjusted according to the Centers for Disease Control and Prevention guidelines, including escalation for severe disease and continuation of suppressive therapy.^2^

## Discussion

Classic skin biopsy findings for HSV infection include balloon degeneration, acantholysis, and viral cytopathic changes, including multinucleated giant cells, chromatin molding, and margination.^3^ Although it is not uncommon to find leukocytoclastic vasculitis in herpes virus infection on histopathologic examination,^4^^,^^5^ there is limited clinical documentation of disseminated cutaneous vasculitis. Several pathogenetic mechanisms have been postulated to explain how herpes viruses induce vasculitis, mainly reported for cases of varicella-zoster virus infection. These include hematogenous spread of viral infection destroying small cutaneous vessels, and direct invasion of the virus to dermal vessels from vesicles or from adjacent nerves.^5^ Given the extensive involvement in our patient, vasculitis secondary to viremia is the most reasonable mechanism to explain her presentation.

Cutaneous HSV infection typically presents as grouped vesicles on erythematous bases that may subsequently develop into erosions or herpetic ulcers. One of the common presentations of disseminated cutaneous HSV infection, regarded as eczema herpeticum, could be seen in patients with a preexisting skin disease that had resulted in an impaired functional barrier.^6^ Rare manifestations, including maculopapular eruption without the presence of vesicles or pustules and diffuse, ulcerative rash, have been reported, which should be differentiated from other causes, such as Stevens–Johnson syndrome, drug reaction with eosinophilia and systemic symptoms, and acute HIV exanthem.^6^

In the reported patient, palpable purpura was distributed over her chest, back, buttocks, and extremities. The erosions and ulcers at the intergluteal folds were considered to be the primary site of recurrent HSV infections, while the virus later disseminated and presented as vasculitis at other sites. As the skin biopsy from the right knee, far from and distinct from the typical primary HSV infection site, revealed concurrent HSV infection and vasculitis pathologically, HSV-induced cutaneous vasculitis should be considered. This causative relationship could be further substantiated by the dramatic improvement of all skin lesions after administration of antiviral treatment and discontinuation of most immunosuppressants. To our knowledge, there is only 1 other report of both clinically and pathologically proven concomitant herpes simplex infection and disseminated vasculitis.^7^ Several reports of dermatomal herpes zoster infection associated with concurrent or subsequent localized vasculitis have also been documented.^5^^,^^8^, ^9^, ^10^ However, HSV infection presenting with generalized vasculitis is rare. Our case provides an unusual presentation of disseminated HSV infection as cutaneous vasculitis in an immunocompromised patient, highlighting the necessity to consider HSV infection as a differential diagnosis when encountering widespread cutaneous vasculitis.

## Conflict of interest

None.
